# Experimental investigation of geochemical and mineralogical effects of CO_2_ sequestration on flow characteristics of reservoir rock in deep saline aquifers

**DOI:** 10.1038/srep19362

**Published:** 2016-01-20

**Authors:** T. D. Rathnaweera, P. G. Ranjith, M. S. A. Perera

**Affiliations:** 1 Department of Civil Engineering, Monash University, Building 60, Victoria 3800, Australia

## Abstract

Interactions between injected CO_2_, brine, and rock during CO_2_ sequestration in deep saline aquifers alter their natural hydro-mechanical properties, affecting the safety, and efficiency of the sequestration process. This study aims to identify such interaction-induced mineralogical changes in aquifers, and in particular their impact on the reservoir rock’s flow characteristics. Sandstone samples were first exposed for 1.5 years to a mixture of brine and super-critical CO_2_ (scCO_2_), then tested to determine their altered geochemical and mineralogical properties. Changes caused uniquely by CO_2_ were identified by comparison with samples exposed over a similar period to either plain brine or brine saturated with N_2_. The results show that long-term reaction with CO_2_ causes a significant pH drop in the saline pore fluid, clearly due to carbonic acid (as dissolved CO_2_) in the brine. Free *H*^+^ ions released into the pore fluid alter the mineralogical structure of the rock formation, through the dissolution of minerals such as calcite, siderite, barite, and quartz. Long-term CO_2_ injection also creates a significant CO_2_ drying-out effect and crystals of salt (*NaCl*) precipitate in the system, further changing the pore structure. Such mineralogical alterations significantly affect the saline aquifer’s permeability, with important practical consequences for the sequestration process.

Due to their wide availability, deep saline aquifers show great promise for the sequestration of carbon dioxide (CO_2_), and therefore the effective mitigation of anthropogenic emission of CO_2_ into the atmosphere^1^,^2^. However, this sequestration brings long-term interactions between CO_2_, rock, and pore fluid (brine), causing unpredictable hydro-mechanical behaviour in the reservoir rock and uncertain long-term stability. In particular, changes in rock permeability critically affect the safety and efficiency of the sequestration process. Unexpected increases in reservoir permeability may cause sudden leakages, harmful to the environment and to human communities; they may also strengthen gradual migration of the CO_2_ plume, allowing it to reach poorly characterized areas that are at even greater risk of leakage. Such considerations have made the effects on reservoir rock permeability a topic of great research interest^3^,^4^,^5^,^6^,^7^, but most studies have focused only on the mineralogical and geochemical consequences^8^,^9^,^10^, with little attention to how reservoir flow is influenced. The impact on flow in deep saline aquifers can only be appreciated by the combined investigation of geochemical and permeability changes with CO_2_ sequestration.

A small number of experimental studies have set out to identify the combined geochemical and permeability effects^11^,^12^, but their reach is limited. For example, Shiraki and Dunn^11^ conducted a hydro-chemical laboratory experiment on dolomite- and anhydrite-cemented Tensleep sandstone saturated with a mixture of CO_2_ and brine for nearly one week, to understand the influence on permeability. They found a reduction in permeability after one week of saturation, arising from the formation of kaolinite in pore throats and other mineral reactions such as the dissolution of dolomite and *K*-feldspar. Muller *et al.*^6^ performed a combined hydro-chemical experiment on Berea sandstone by flushing *NaCl* saturated cores with dry CO_2_, and found a reduction in permeability due to the precipitation of halite minerals. Experiments on calcite- and dolomite-cemented sandstone by Ross *et al.*^12^ showed a permeability increase in the reservoir formation from the dissolution of carbonate minerals; they report this increase as being due to enhanced pore space modification, associated with the dissolution of carbonate cement bonds. However, all these experiments were limited to short-term effects (2–4 weeks), revealing nothing about flow under long-term CO_2_ exposure. This is a serious gap in our knowledge. In the long term, carbonic acid from CO_2_ in brine reacts with many minerals in the formation, such as calcite, siderite, dolomite, quartz, barite, muscovite, feldspar, and clay minerals^3^,^6^,^7^,^8^,^12^,^13^,^14^,^15^. These reactions dissolve rock cementation, weakening grain bonds and altering flow characteristics in ways that compromise the efficiency and safety of the whole sequestration exercise. In fact, any correct identification of alterations from injected CO_2_ demands a rigorous and systematic comparison, with a bench study of inert gas injected into reservoir rock under the same conditions. That comparison would eliminate the effects on rock pore structure from gas injection generally, such as pore expansion and shrinkage. However, no such comparison has been undertaken in previous combined geochemical and permeability studies.

The present investigation is driven by that demand. It is a comprehensive, combined experimental study of chemical, mineralogical, and permeability alterations in saline aquifers from reactions in long-term CO_2_ sequestration, over a realistic duration of 1.5 years. The study aims especially to provide the parameters needed for reservoir modelling of CO_2_ sequestration. By allowing sufficient time for interactions to run their course, mineral reactions can emerge that are never seen in short-term saturation. The investigation will therefore be highly relevant to our understanding of actual flow properties in reservoir rock under sequestration conditions. The selected conditions for CO_2_ reactions, i.e. a pressure of 10 MPa and a temperature of 40 °C, represent real field behaviour with CO_2_ in its super-critical state. Permeability tests were included for a wide range of injection pressures (2–6 MPa) and confining pressures (10–30 MPa) to simulate field conditions accurately. This innovation enables the first-ever evaluation of the effective stress fields induced by rock mineral alterations. Care has been taken to isolate the pure CO_2_ reaction effects on chemical, mineralogical, and flow behaviour in reservoir rock, by comparing them with alterations brought on by nitrogen (N_2_), which is chemically inert for the present purposes.

## Methods

### Sample description

Brine-saturated Hawkesbury sandstone samples were used to represent the saline aquifer formation, and their possible geochemical and mineralogical alterations upon CO_2_ injection were investigated. The sandstone samples were collected from the potential Gosford site for carbon capture and storage (CCS) in the Sydney basin, a formation belonging to the early Triassic period. Their composition is mainly quartz, calcite, and kaolinite, according to X-ray diffraction (XRD) analysis (by weight, around 60% quartz, 26% calcite, 6% kaolinite, 5% barite, 1% siderite, 1% muscovite, and 1% other clay minerals such as illite and smectite). It may be considered a carbonate-cemented sandstone formation, as it contains high percentages of calcite minerals in the pore structure.

Most current field-scale CO_2_ sequestration projects have used saline aquifers with sandstone host rocks, because preferable saline aquifers for CO_2_ sequestration require adequate permeability and porosity values for successful CO_2_ injection and storage. Among the possible sandstone formations, carbonate-cemented sandstone formations with more than 20% carbonate minerals have more preferable characteristics for CO_2_ sequestration, due to their potential to trap greater amounts of CO_2_ through carbonate reactions^7^. This was the main reason for using Hawkesbury sandstone in this study, since Hawkesbury sandstone has a high percentage of calcite minerals (26%) and many favourable characteristics for CO_2_ storage process in terms of its hydro-mechanical and mineralogical properties. A comprehensive description of the mineralogical, chemical and geo-mechanical information of Hawkesbury sandstone is given in Table 1.

The Hawkesbury formation consists of an un-layered, white-grey coloured, and medium-to fine-grained rock mass that exhibits a mostly inhomogeneous structure. Therefore, great care was taken to obtain homogeneous samples from this formation, and only core specimens without visible discontinuities were selected for the experiment.

### Sample preparation and reaction process

Hawkesbury sandstone blocks were collected and cored according to the ISRM standards in the Deep Earth Energy Laboratory (DEEL), at Monash University. The sample diameter was selected to be 38 mm and the cored samples were cut into 76 mm-long cylinders. The two ends of the samples were carefully ground to create smooth parallel faces, and the prepared specimens were oven-dried for 24 hours under 40 °C (a low temperature was selected to avoid possible thermal cracking) before beginning the reaction process.

Three different reaction conditions were selected: pure-brine-reacted (without any gas injection), brine+CO_2_-reacted, and brine+N_2_-reacted. Samples were first saturated with brine at a 20% *NaCl* concentration (% by weight) in desiccators under vacuum (0.2 MPa suction pressure). The samples were weighed at regular intervals during the saturation process, and when full saturation was reached, 15 samples were left inside the desiccator without applying vacuum for 1.5 years, to allow time for interaction. Later, this set was used as pure brine-reacted samples for both chemical and permeability measurements. The remaining 15 samples were removed from the desiccators and kept in reaction chambers to achieve CO_2_- and N_2_-reacted conditions. Before brine-reacted samples were placed in them the reaction chambers were filled with brine of the same concentration (20%), and samples were then inserted to facilitate the CO_2_ and N_2_ reactions. Two separate chambers were used for CO_2_ and N_2_ injection, and both gases were injected at 10 MPa pressure at 40 °C for 1.5 years to obtain CO_2_+brine- and N_2_+brine-reacted sandstone samples.

In the present study, a reaction period of 1.5 years was investigated, based on the time required to complete the reaction of existing major rock minerals (quartz, calcite and kaolinite) with CO_2_ and brine to identify the ultimate alterations of these major rock minerals upon CO_2_ interaction and the corresponding aquifer flow response. It is known that different rock minerals need different timescales and degrees of disequilibrium to complete their reaction with CO_2_ and brine^16^. The time required to create the equilibrium of the resulting buffer solution (due to initial dissolution of CO_2_ in brine) is within 1 to 2 years of interaction under reservoir conditions^17^. If the time required for mineral dissolution is considered, according to Knauss and Wolery^18^ and Davis *et al.*^19^, the initiation of the quartz reaction with CO_2_ and brine requires a considerable geological time, which is certainly more than 1 year and kaolinite mineral also requires a considerable geological time-frame to initiate the early reaction. Therefore, conducting short-term experiments fails to identify such reaction-creating influence. As a result, considering the time-frame available for the study, 1.5 years was selected as being a reasonable time for the CO_2_/brine/rock mineral interaction in this study. However, even using a 1.5-year time period, it is not possible to capture all the possible rock minerals alterations that occur with CO_2_ and brine interaction (e.g. precipitation of feldspar and secondary precipitation of calcite and quartz). Therefore, only the dominant reactions, such as the initial dissolution of quartz, calcite, kaolinite, barite, and siderite and the salt drying-out effect were considered in this study. However, it should be noted that the reaction of some rock minerals with CO_2_ and brine can occur within a very short time period. For example, carbonate mineral reactions, including calcite, magnesite and siderite, may occur within 2–4 weeks of interaction with CO_2_ and brine^6^,^7^,^8^ and therefore can be captured in even short-term experiments.

### Permeability tests

A series of high-pressure tri-axial permeability tests was conducted on the prepared variously- reacted sandstone samples (pure brine, brine+CO_2_, brine+N_2_), under undrained conditions. For the present study, three replicates were used in each test condition and permeability evaluation was performed taking its mean value (with standard deviation 1–3%). Details of the high-pressure tri-axial set-up and the sample assembly procedure can be found in Rathnaweera *et al.*^20^. The permeability tests were performed by injecting CO_2_ into the various prepared sandstone samples, and the corresponding downstream pressure developments were recorded to find the CO_2_ permeability under each test condition using the pressure decay approach. Each sample was first kept inside the high-pressure cell; after assembly, the required confinement was applied under constant temperature and gas injection was initiated while recording the downstream pressure development. A high-precision syringe pump was used to inject CO_2_ into the sample at constant injection pressure (2–6 MPa) under the required confinement (between 10 and 30 confining pressures were considered, to simulate the reservoir depth effect).

Just before the permeability tests, the reacted sample was removed from the reaction chamber and inserted into the tri-axial cell. The brine inside the samples was removed by injecting CO_2_ at 1 MPa injection pressure under 10 MPa confining pressure, and the corresponding flush-out brine weight was measured over time using an accurate balance. This process was performed until brine removal ceased (at which time the measured weight value becomes constant). After confirming that there was no mobile brine inside the sample (there would be brine held in place by capillary forces), the normal permeability tests were initiated for single-phase CO_2_ flow behaviour inside the sample. Here, the purpose of continuing the three kinds of reactions for 1.5 years was to provide sufficient time to initiate CO_2_/brine/rock mineral interactions and for the sample pore structure to be changed accordingly. Permeability tests on these altered samples gave an opportunity to see how the sample flow characteristics had changed following these interactions.

Permeability tests were initiated using brine-reacted samples after removing their mobile brine. CO_2_ was injected at 2 MPa injection pressure under 10 MPa confining pressure, and the downstream pressure development was recorded. Once it became constant, the developed pressure was released by opening the downstream valve at the extremely slow rate of 0.02 MPa/s to avoid any damage to the sample’s pore structure. After this release, the downstream valve was closed and the experiment proceeded to the second stage of CO_2_ injection (at 3 MPa) under the same confining pressure, and similar permeability tests were performed for a series of injection pressures (3, 4, 5, and 6 MPa). Once the permeability tests were completed with confining pressure set to 10 MPa, it was increased to the next level (first to 15 MPa confining pressure; then to 20, 25, and 30 MPa) and the permeability tests were conducted for the same injection pressures. All the brine-reacted, brine+CO_2_- and brine+N_2_-reacted samples were similarly tested, having first removed the mobile brine, for the same series of injection and confining pressure conditions.

### Chemical and mineralogical analysis

#### Inductively-coupled plasma mass spectroscopy (ICP-MS) and inductively-coupled plasma atomic emission spectroscopy (ICP-AES)

ICP-MS and ICP-AES, two advanced analytical techniques for elemental determinations, were used in this study to examine the trace and ultra-trace elements^21^ of the brine samples taken from the reaction chambers and desiccators after the 1.5-year reaction period. The main purpose of these chemical analyses was to identify changes in pore fluid properties due to CO_2_/brine/rock mineral interactions. Table 2 shows the operating conditions for the ICP-MS and ICP-AES tests. A model SPQ 8000A instrument coupled with a quadrupole-type spectrometer was used for the ICP-MS tests, and a model plasma atom comp MK11instrument was used for the ICP-AES tests.

#### Scanning electron microscopy (SEM) analysis

A detailed SEM analysis was also conducted to identify mineralogical changes in reservoir rock pore structures after three kinds of interaction. Samples reacted with brine+CO_2_ and brine+N_2_ were collected from the reaction chambers, and pure-brine-reacted samples were taken from the desiccator. SEM analysis was also performed on natural samples to identify the natural condition of the rock microstructure and compare it with the saturated samples’ microstructures. A rock slice around 1 mm thick was prepared for each condition and a 3 μm titanium coating was applied before SEM testing, to avoid a charging effect during the image-scanning process. Tests were carried out under wet conditions for brine+CO_2_-, brine+N_2_-, and pure-brine-reacted samples, and dry conditions for natural samples. An FEI Nova Nano SEM machine coupled with two Brucker EDS and in-lens detectors was used in low-vacuum mode to capture changes in the sandstone microstructure. Furthermore, a spot size of 3.5 and a magnification of 10,000× were used to analyse the microstructure of the sandstone specimens under each reaction condition.

## Results

### Interacting brine, CO_2_, and rock produced mineralogical and geochemical alterations

Three differently-reacted pore fluid conditions brine/rock (plain brine, without gas), brine/CO_2_/rock, and brine/N_2_/rock were analysed using ICP-MS, ICP-AES, and SEM techniques, and a plain unreacted brine sample was also tested as a control. The brine solution under each condition represented actual pore fluid in a saline aquifer. Dissolved 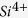, 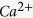, 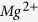, 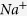, 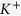, 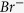, 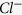 and 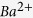 were determined using the ICP-AES method, and 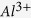, 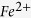, and 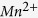 using the ICP-MS method. Results of the desiccator and the chamber analyses are reported in Table 3. As the table shows, there is a significant pH drop in the brine+CO_2_-reacted solution of around 49% (7.41 to 4.81) after long-term CO_2_ injection, but no such drop in the brine+N_2_-reacted solution. The observed pH reduction is therefore believed to be from the dissolution of injected scCO_2_ in brine, resulting in the creation of an acidic medium: carbonic acid in the pore fluid, as shown in Eq. [1].


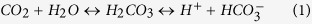


As expected, this pH reduction occurred only in the brine+CO_2_-reacted solution, confirming the comparatively reactive nature of injected CO_2_. Past studies have emphasized the relevance of this CO_2_ dissolution in brine for the storage of injected CO_2_ to solubility trapping in deep saline aquifers^22^. However, this dissolution process is not only important for solubility trapping; it also greatly affects the mineral-trapping process, because free 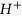 ions released into the pore fluid may react with minerals in the formation, dissolving them and eventually changing the composition and structure of the reservoir rock. Since such alterations mainly occur in silicate- and carbonate-cemented grain-to-grain contacts, they are more evident in formations where these minerals predominate. Studies have shown that carbonate-cemented sandstone in deep saline reservoirs yields the required geological conditions for CO_2_ sequestration, and more than 60% of petroleum reservoirs are carbonate reservoirs^23^.

ICP-AES and ICP-MS chemical analyses revealed the leachability of some minerals, when ions move from the sample into aquifer pore fluid as a result of these dissolution reactions. As mentioned previously, calcite dissolution is one of the most important dissolution reactions that occur during sequestration. Generally, the increment of 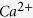 ion concentration in the pore fluid (compared to its initial stage) provides basic identifying evidence for the calcite mineral dissolution process, which can be further investigated by microstructural analysis using SEM.

**Figure 1:**
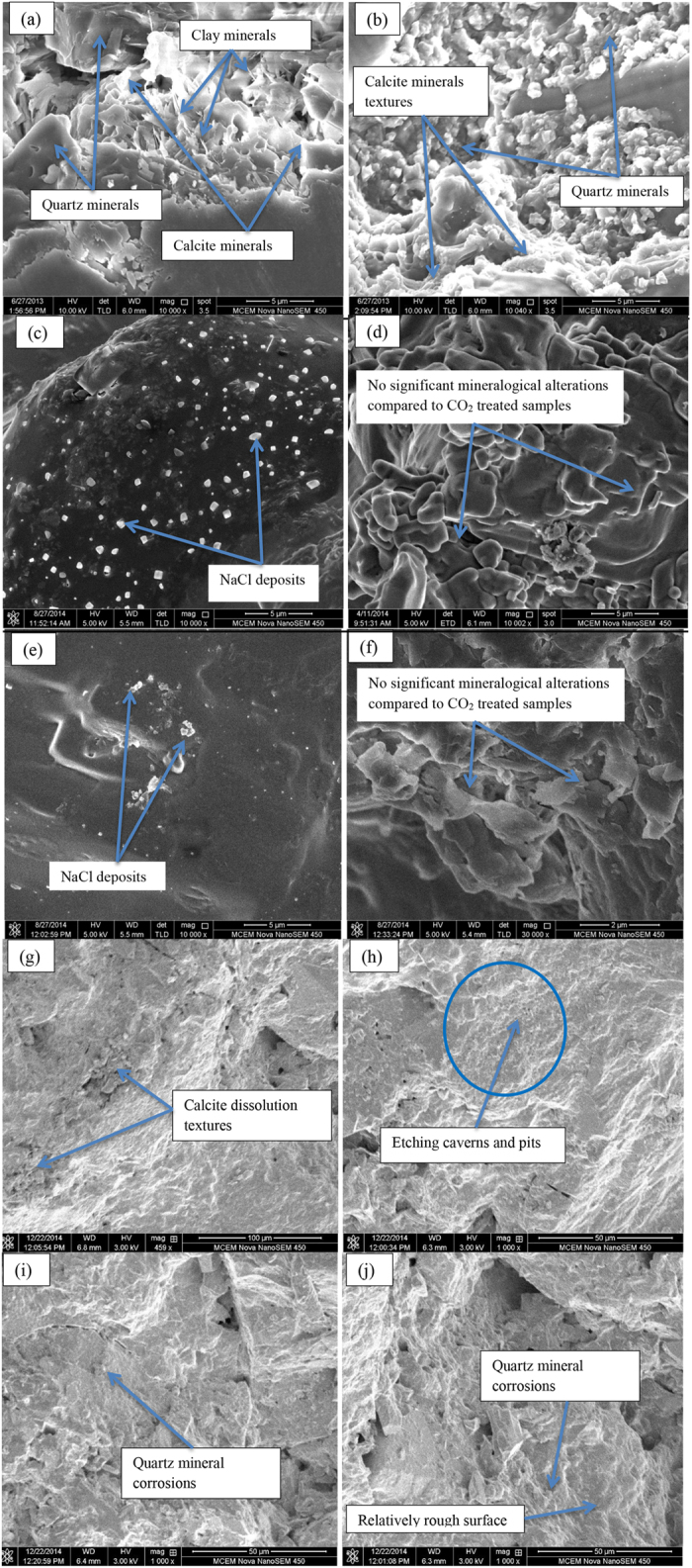
Results of SEM analysis (**a**,**b**) natural sample (**c**,**d**) brine (without gas reaction) (**e,f**) brine+N_2_-reacted samples and (**g**–**j**) brine+CO_2_-reacted sample.

According to the ICP-AES analysis, significant quantities of 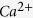 ions leached into the pore fluid from the samples reacted with brine+CO_2_, compared to either pure brine or brine+N_2_. The initial concentration of 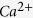 ions in pure brine was 100.1 mg/l. This increased to around 482.7 mg/l after introducing the sandstone sample into the brine, up to around 440.3 mg/l after introducing N_2_+sandstone, and up to around 3237 mg/l after introducing CO_2_+sandstone. The presence of CO_2_-releasing 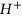 clearly accelerates calcite dissolution. SEM analysis confirmed this much greater effect, showing a significant calcite dissolution texture in brine+CO_2_-reacted samples compared to the other samples. Figure 1(a,b) show the mineral structure of a natural sample, and Fig. 1(g–j) show the mineral structure of brine+CO_2_-reacted samples. The SEM image of a natural sample (Fig. 1(a,b)) shows the initial calcite mineral texture in the reservoir rock mass pore structure before significant changes caused by the CO_2_ interaction (Fig. 1(g–j)). The SEM images of brine+CO_2_-reacted samples (Fig. 1(g–j)) exhibit the dissolution textures of calcite minerals, with a relatively rough surface after CO_2_-brine-rock interaction compared to a smooth surface in the natural sample. According to Marbler *et al.*^7^ and Gledhill and Morse^23^, the dissolution of calcite in carbonate-cemented reservoir rocks significantly alters the arrangement of pores. It also helps create secondary pores by changing the effective stress and flow characteristics of the formation, and the combined effect is permeability enhancement in the reservoir and caprock. Although this heightened permeability increases CO_2_ injectability into the reservoir and improves the aquifer’s storage capacity, the increase in reservoir permeability brings a greater risk of CO_2_ back-migration into the atmosphere.

Moreover, the ICP-MS results for brine+CO_2_-saturated samples also showed some enrichment patterns in 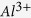, 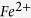, and 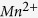 ions (see Table 3) compared to those in pure brine; and these patterns were not observed in samples reacted with pure brine or brine+N_2_. The accumulated ions in brine+CO_2_-reacted pore fluids are due solely to CO_2_. The increased 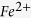 concentration is believed to be related to the dissolution of siderite minerals from the rock mass, and the increase in 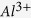 and 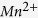 ions is probably from dissolution of clay minerals such as smectite, illite, kaolinite, and muscovite.

Apart from these mineral dissolutions, topographic SEM images of brine+CO_2_-reacted samples (Fig. 1(h)) display tiny rectangular etching caverns and pits inside the pore structure, which do not appear in the other samples. These are thought to come from barite minerals dissolved from the rock mass due to CO_2_ exposure, again confirming the effect of long-term injection of CO_2_ on reservoir mineral structure.

Significant quartz mineral corrosion was also found, in the chemical analyses. 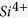 concentration in the pore fluid was considerably increased after the introduction of CO_2_, most likely from dissolution of quartz in the rock mass. According to the ICP-AES analysis, the 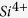 ion concentration increased from 0.11 to 4118 mg/l with CO_2_ reaction and to 815.31 mg/l with N_2_ reaction. This quartz corrosion in the vicinity of CO_2_ is also confirmed by the SEM images of the brine+CO_2_-reacted samples (see Fig. 1(i,j)), consistent with the findings of Kaszuba *et al.*^22^, who found similar patterns in quartz minerals with CO_2_ introduced into brine-saturated sandstone. Marbler *et al.*^7^ provide valuable explanations of how quartz corrosion affects the structure of rock pores: the dissolution of primary and secondary silicate mineral rims around the quartz-cemented grains reduces the strength of quartz grain-grain contacts, significantly changing the rock mass pore structure. The small quartz mineral dissolution observed in brine+N_2_- and pure-brine-reacted samples (both negligible compared to the brine+CO_2_-reacted sample) is attributed to a slight corrosive effect of brine solution itself.

**Figure 2:**
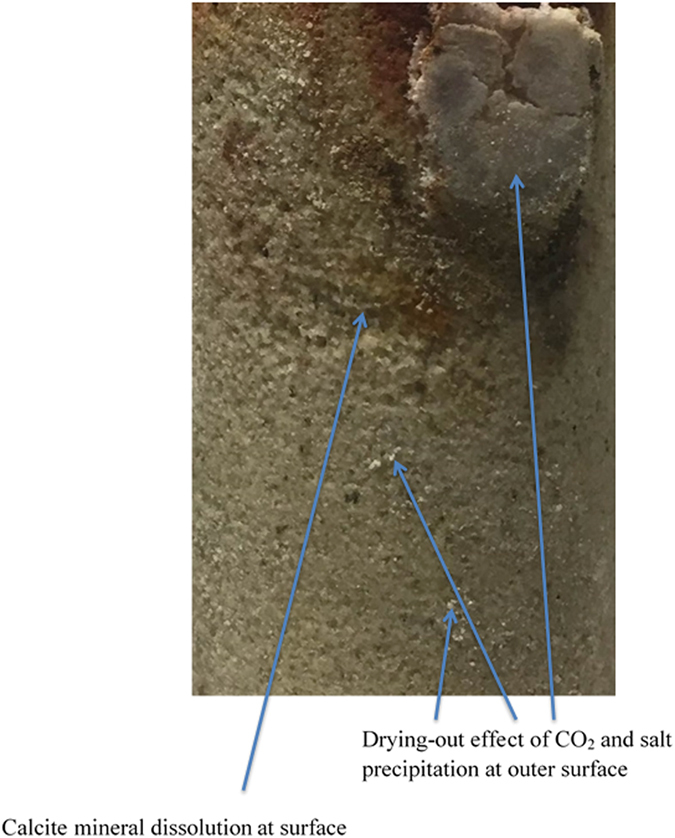
Results of visual inspection of the brine+CO_2_-reacted sample; deposits of NaCl crystals (salt precipitation) and calcite dissolution textures at the outer surface of the sample.

Apart from mineral dissolution, chemical analyses uncovered a significant CO_2_ dry-out effect and *NaCl* crystal precipitation in the rock mass pore system in brine+CO_2_-reacted samples. This is consistent with the findings of Pruess and Muller^24^, who explained that the injection of CO_2_ into saline aquifers causes formation dry-out and salt precipitation near the injection well, reducing porosity, permeability, and injectivity in the formation. When scCO_2_ is injected into a saline aquifer, water evaporates into the scCO_2_-phase, leaving the remaining brine with a higher salt concentration. If this concentration is greater than the solubility of salt under the prevailing pressure and temperature conditions, salt will precipitate; so salt precipitation is clearly associated an increase in salinity (*Na*^+^ concentration). According to the ICP-AES results, 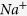 in the initial brine sample increases from 191,433 to 277,863 mg/l with CO_2_ injection, thought to be related to salt precipitation. This was confirmed by SEM analysis, according to which the brine+CO_2_-reacted samples display some *NaCl* crystal depositions inside the rock pore structure. Visual inspection of the brine+CO_2_-reacted samples confirmed displacement of brine from the sample by injected CO_2_ and there were consequent deposits of *NaCl* crystals at the outer surface of the samples (see Fig. 2). Interestingly, the SEM results for samples reacted with either plain brine or brine+N_2_ also showed a small amount of *NaCl* deposition inside the pore structure (see Fig. 1(e)). According to the ICP-AES analysis, 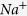 concentration from 191,433 to 198,304 mg/l for plain brine samples and from 191,433 to 195,917 mg/l for brine+N_2_ reacted samples. The 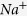 concentration increase in plain-brine-reacted samples is therefore greater than in brine+N_2_-reacted samples, and considerably less than in brine+CO_2_-reacted samples. In addition to 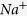, slight increments in 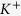 and 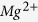 content (compared to the initial brine solution) were observed in all the reaction conditions, presumably related to salt precipitation from the pore fluid. The 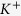 and 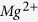 content increments are more significant in brine+CO_2_-reacted samples than in all other samples (Table 3), probably due to the greater salt precipitation from the CO_2_ dry-out effect.

According to Table 3, it is clear that significant rock mineral alterations are caused by CO_2_ injection, leading to changes in the chemical and mineral structure of the rock mass. To facilitate further understanding of this CO_2_-related dissolution process, the amounts of dissolved rock minerals were calculated as percentages of the initial compositions of each rock mineral in natural samples. Table 4 displays the calculated percentage values of each dissolved rock compound. According to Table 4%, 1.11%, and 1.21% of calcite was dissolved in brine+CO_2_, brine+N_2_, and plain-brine-reacted samples respectively, compared to an initial measure of 39.73 g (26% of total rock mass). Moreover, 4.49%, 0.88%, and 0.95% of quartz was dissolved in brine+CO_2_, brine+N_2_ and brine-reacted samples respectively, compared to an initial measure of 91.68 g (60% of total rock mass). As Table 4 shows, significant dissolution of barite mineral occurred in brine+CO_2_-reacted samples (10.79%) compared to the other two saturation conditions, with 0.16% and 0.17% of its initial barite mineral composition being dissolved in brine+N_2_ and brine-reacted samples respectively. The calculated dissolved mineral percentage values based on the initial composition indicate significant effects of CO_2_ saturation on the structure of reservoir rock minerals compared to the other two tested conditions. Table 4 also reveals that this CO_2_ injection-induced mineral alteration process (mineral trapping) is truly a long-term phenomenon; it dissolved only between 4% and 10% of the total mineral composition over 1.5 years of reaction in the present study. Significantly more dissolution can be expected over the large time-scales (more than 100 years) that are relevant in practice. This argument is consistent with previous research by Ranganathan *et al.*^25^.

### Flow characteristics affected by rock alteration in reservoir formations

**Figure 3:**
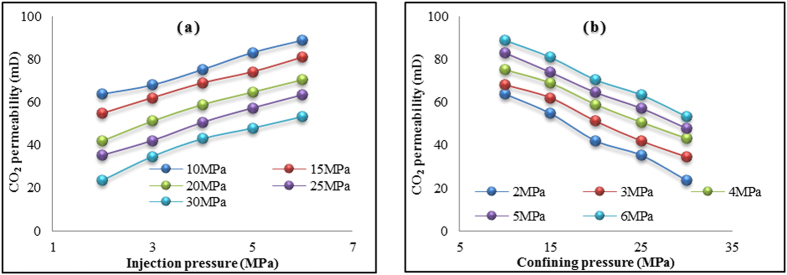
CO_2_ permeability variation in brine+CO_2_-reacted sample (**a**) with injection pressures and (**b**) with confining pressures.

Variation of flow characteristics in reservoir formations during and after CO_2_ injection is one of the critical challenges for CO_2_ sequestration in saline aquifers. Alterations in the permeability and porosity of formations during long-term injection consequent upon interactions of minerals, brine, and CO_2_ have been widely reported in field-scale studies^13^,^14^. The corresponding permeability of the sample under each injection condition was determined using the pressure decay approach^26^,^27^. According to Pan *et al.*^27^, pressure decay curves can be modelled as Eq. [2]:


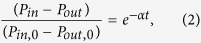


where 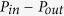 is the pressure difference between the gas inlet and outlet measured by a differential pressure transducer, 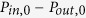 is the initial pressure difference between gas inlet and outlet, *t* is the time and *α* is as given in Eq. [3]:


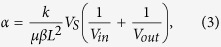


where *k* is the permeability, *μ* is the flowing gas viscosity, *β* is the gas compressibility, *L* is the sample length, 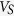 is the sample volume, and 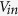 and 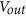 are the volume of the gas inlet and outlet plumbing systems. The effect on CO_2_ permeability of pore structure modifications created by brine–CO_2_ interactions in the tested sandstone was investigated for five injection pressures (from 2 to 6 MPa) under five confining pressures (from 10 to 30 MPa), at 40 °C. Figure 3 shows the calculated CO_2_ permeability values; the CO_2_ injection pressure’s influence on permeability is shown in Fig. 3(a), and the confining pressure’s influence on permeability is shown in Fig. 3(b).

As expected, sample permeability increases with increased injection pressure and reduced confining pressure, both of which are mainly related to the effective stress, which is lowered by increasing the injection pressure or by reducing the confining pressure applied to the sample^2^,^20^. For instance, increasing injection pressure from 2 to 6 MPa under 20 MPa confining pressure raises the sample permeability by around 67%, and increasing the confining pressure from 10 to 30 MPa lowers it by around 43%.

**Figure 4:**
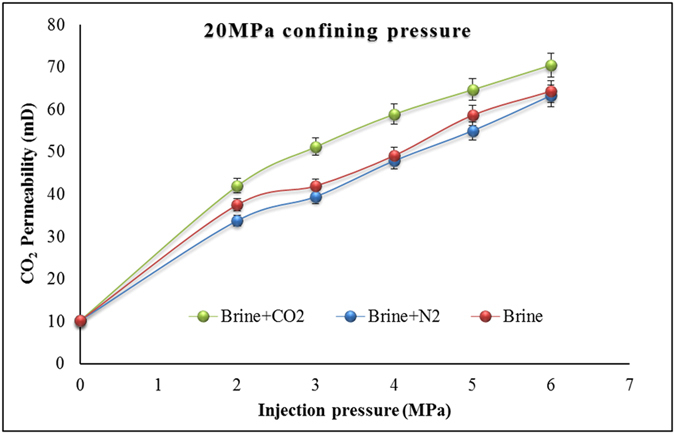
Permeability variations for each reacted condition under 20MPa confining pressure.

The main purpose of this study, however, was to identify the effect on sample permeability of interactions between minerals, brine, and CO_2_ in rock. Therefore, CO_2_ permeability values for brine+CO_2_-reacted samples were compared with CO_2_ permeability through brine+N_2_ and brine-only samples. Figure 4 shows the calculated CO_2_ permeability values under 20 MPa confining pressure for each saturation condition. According to the figure, rock minerals-brine-CO_2_ interaction has a significant effect on CO_2_ permeability in the reservoir rock samples, and CO_2_ permeability through brine+CO_2_-reacted samples is clearly higher than through brine+N_2_ and brine-only samples. For example, at 4 MPa injection pressure and 20 MPa confining pressure, CO_2_ permeability through brine+CO_2_-reacted samples is around 17% and 19% higher than through brine-only and brine+N_2_ samples, respectively. The permeability enhancement in the brine+CO_2_ samples clearly reveals the effect of injected CO_2_/brine/rock interactions on the flow characteristics of deep saline aquifers and its influence on rock mineralogical alterations.

Permeability was found to be essentially the same through the brine+N_2_-reacted samples and the plain-brine-reacted samples, confirming the negligible influence of N_2_ saturation on sample flow characteristics. This is because, since non-reactive N_2_ causes no mineralogical or chemical reaction in the sample during saturation, its pore structure is not noticeably altered, even over 1.5 years. This is further confirmed by the results of the SEM and ICP analyses. Sandstone permeability enhancement with CO_2_ injection must therefore be seen as related to the reactions between rock minerals and carbonic acid produced by the interaction of CO_2_ and brine. This conclusion is reinforced by the results of ICP-AES analysis, where the collected chamber pore fluid showed significant silicate and calcite mineral dissolution from the sandstone sample, and also by the SEM images, which clearly exhibit alterations in rock cement from CO_2_ reactions. The dissolution of pore-filling calcite and calcite coatings of detrital minerals changes the pore structure and consequently the porosity of the reservoir rock, eventually creating new pathways for CO_2_ migration and enhancing the permeability characteristics of the reservoir formation.

**Figure 5:**
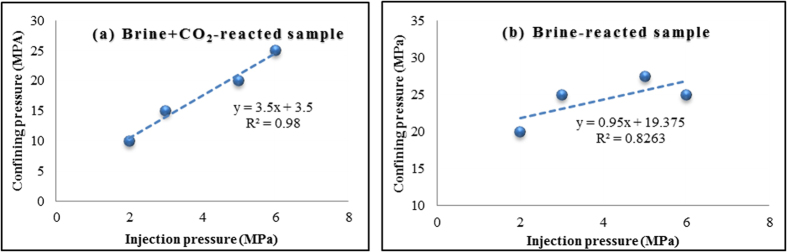
Variation in effective stress coefficient for samples: (**a**) brine+CO_2_-reacted; and (**b**) pure brine-reacted.

Weakening of the rock mass mineral grains with CO_2_ injection-induced chemical and mineralogical changes also affects the effective stress patterns acting on the pore space system. It was therefore important to assess the impact of the effective stress field on permeability during brine/rock/CO_2_ interaction and the variation of the effective stress coefficient for permeability under various conditions. The effective stress coefficient for the variation of permeability can be obtained from iso-permeability lines drawn as a function of pore pressure and confining pressure, and the slope of each curve gives the effective stress coefficient^28^. Figure 5 shows the iso-permeability lines for the brine+CO_2_ and brine-only samples, which give respectively 3.5 and 0.95 effective stress coefficients. The introduction of CO_2_ into brine-saturated rock samples has therefore significantly raised the effective stress coefficient. Such observations indicate the importance of precisely understanding the possible consequences of rock hydro-mechanical changes as a result of mineralogical changes during the CO_2_ injection process for any CO_2_ sequestration field project.

## Discussion

Although the laboratory experimental results provide crucial evidence related to mineralogical rock alterations in deep saline aquifers, modelling of reservoir simulations, coupled with both geochemical and geophysical behaviours, are also important to understand the reaction mechanisms of rock minerals upon exposure to CO_2_/brine during real field time-frames due to the practical difficulties of laboratory conditions, particularly time limitations. However, the precise simulation of such mechanisms requires correct identification of potential reaction mechanisms and their behaviour over the time of CO_2_/brine interaction and their variation with surrounding factors (CO_2_ partial pressure, reservoir temperature and pH). To date, most related laboratory experiments have been conducted over short-term time durations, and they have therefore failed to capture long-term reaction mechanisms.

Among the various possible reaction mechanisms, if the possible short-term mechanisms are first considered, calcite dissolution is predominant. According to the existing findings, the calcite dissolution process mainly involves three simultaneous reaction mechanisms, as given below^29^;


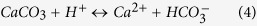



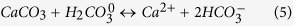



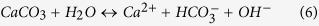


These reactions are dependent on the CO_2_ partial pressure, reservoir temperature and pH, and the calcite dissolution rate increases with increasing CO_2_ partial pressure and decreasing pore fluid pH^29^, and varies with reservoir temperature^30^ and foreign ions (e.g. orthophosphate)^31^. The short-term experimental study conducted by Wigand *et al.*^17^ found an enhancement of calcite dissolution over time (after five days of CO_2_ interaction) with associated reduction of pH and the reduction of the calcite reaction rate after a certain time (two months) with the initiated pH increment. In this present study it was not possible to calculate the calcite dissolution rate over time. However, the long-term interaction of CO_2_/brine/ rock caused a significant calcite dissolution, as evidenced by the observed 100.1 mg/l to 3237 mg/l *Ca*^2+^ concentration increment in the pore fluid. Since CO_2_ geological sequestration is conducted over long time-scales in the field, according to this study, there is a greater possibility of enhancing the calcite dissolution rate with the CO_2_/brine interaction.

In relation to possible long-term reactions, the quartz mineral reaction dominates the mineral trapping process of CO_2_ in deep saline aquifers, as the reservoir rocks are generally abundant in quartz minerals. The general reaction mechanism of quartz is given bellow:


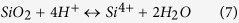


According to existing studies, the quartz dissolution rate reduces with increasing pore fluid pH and increases with the presence of cations in the pore fluid (e.g. sodium, calcium and magnesium)^18^,^32^,^33^. However, since this quartz dissolution process takes extensive time to initiate, this cannot be identified in short-term laboratory experiments. For example, the short-term experimental work conducted by Wigand *et al.*^17^ did not find any quartz dissolution in their experiments. However, the long-term experiments conducted in the present study revealed that the interaction of quartz with CO_2_ and brine causes significant quartz dissolution, as evidenced by the observed 0.11 to 4118 mg/l 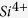 ion concentration in the pore fluid after 1.5 years of interaction.

Apart from the quartz dissolution, kaolinite dissolution is also a dominant long-term reaction mechanism that occur during CO_2_ sequestration in saline aquifers, which mainly affects the reservoir rock mass pore structure by altering grain-to-grain contacts. The basic kaolinite dissolution reaction mechanism in acidic systems is given below:


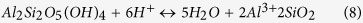


According to the research, the rate of kaolinite dissolution increases with increasing reservoir temperature and pH of the aquifer pore fluid^34^ and varies with the available 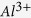 and 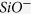 complexes^34^. According to the study conducted by Gunter *et al.*^35^, the final influence of CO_2_/brine interaction on this kaolinite reaction is hard to determine, as the dissolution of *Ca*^*−*^ feldspar can re-precipitate kaolinite minerals during the sequestration process. However, 1.5 years of long-term interaction of kaolinite /brine/CO_2_ in this study clearly caused a significant dissolution of kaolinite mineral, as evidenced by the observed increase of 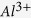 ion concentration in the pore fluid after 1.5 years of interaction. This may have been influenced by the fact that the sandstone samples in this study do not contain feldspar minerals.

In general, the incorporation of laboratory data with field simulations has become one of the major challenges in predicting the long-term fate of mineral reactivity in reservoirs. This is because, reservoir rock mineral dissolution and precipitation may alter the reaction mechanisms over time, which progressively modifies the existing flow pathways for CO_2_ movement. Therefore, it is important to consider these processes, including the coupling between reaction kinetics and mass transport processes when modelling reservoir-scale simulations. Long-term laboratory experiments therefore offer more promising data for the numerical simulation of CO_2_ sequestration.

The effective implementation of CO_2_ sequestration in deep saline aquifers requires significant development in the scientific understanding of mineral reaction-induced reservoir flow property alterations and the corresponding influence on the safety of the process in terms of the effect on caprock integrity and the corresponding possibility of CO_2_ leakage. According to the findings of this study, the significant rock mass mineralogical and pore structures alterations caused by around 1.5 years of long-term CO_2_ interaction with the reservoir may cause its permeability to be enhanced by around 10%. According to Rochelle *et al.*^36^, even such a small alteration in reservoir rock permeability may have a significant effect on the effectiveness of the sequestration process. According to the field-scale observations of Arsyad *et al.*^37^, CO_2_ sequestration created permeability enhancements in Ainoura and Berea sandstone formations, leading to easy movement for CO_2_ plumes to migrate into upper cap rock layers, generating high risk of CO_2_ leakage from the aquifer to surrounding groundwater zones. According to these researchers, enhanced reservoir permeability during the CO_2_ sequestration process in saline aquifers may cause pore pressure enhancement in the aquifer and consequently affect the caprock, creating a negative impact on caprock stability. This is because, if pressure reaches the overburden pressure of the caprock, it may fail, creating hydraulic fractures that will cause CO_2_ leakage into the surrounding aquifers and the atmosphere.

Apart from this, the moving CO_2_ from the aquifer to caprock start to dissolute rock minerals in the caprock^17^. Generally, in deep saline sequestration, mudstone is widely found as a caprock sealing^38^, and contains considerable amounts of clay minerals, quartz and feldspar^39^. Therefore, the presence of these rock minerals has the potential to cause reactions with the dissolved CO_2_ in brine, creating major changes in the caprock structure. Rutqvist and Tasang^39^ confirmed the possibility of reacting these caprock minerals with dissolved CO_2_ in brine and showed that supercritical CO_2_ can react with the organic contents of the caprock including clay minerals and cause considerable changes in the permeability and porosity of the caprock. Moreover, Rochelle *et al.*^40^ also stated that dissolved CO_2_ in brine can react with the overlying caprock, thus reducing the caprock’s sealing properties. Such caprock mineral alterations lead to the formation of new flow pathways along the caprock, creating a leakage risk from the aquifer to surrounding fresh water aquifers and ultimately back-migration into the atmosphere. This is a serious issue and negatively affects the potential implementation of CO_2_ sequestration in deep saline aquifers.

## Conclusions

CO_2_ injection into a deep saline aquifer during the sequestration process causes its hydro-mechanical properties to be significantly altered, and the purpose of this study is to identify how CO_2_ sequestration affects the mineralogical structure and alters the aquifer’s flow response. According to the results of permeability tests on brine-saturated sandstone samples obtained from the Sydney basin, the following conclusions can be drawn:

- ➢ Long-term CO_2_ reaction causes a carbonic acid to form in the aquifer, which causes a significant pH drop in the pore fluid, the observed drop in this study being around 49% after a 1.5 years.
- ➢ Importantly, a huge free 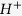 ions release occurs during this acid formation process, which has a significant influence on the aquifer’s mineralogical structure. The 1.5 years of CO_2_ + brine reaction in this study caused a significant dissolution of some rock minerals, including 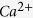, 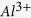, 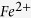, quartz and barite minerals. Of the, 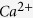 and quartz dissolutions were prominent, and caused the pore fluid 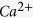 concentration to increase from 100.1 mg/l to 3237 mg/l and the pore fluid 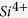 ion concentration to increase from 0.11 to 4118 mg/l.
- ➢ Long-term CO_2_ reaction also creates a significant CO_2_ drying-out effect and NaCl crystallization (salt) in the aquifer’s rock pore space by altering the pore structure. The tests showed a 191,433 to 277,863 mg/l increment in 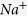 concentration and also considerable enhancements of 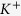 and 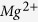 in the pore fluid after the CO2 reaction.
- ➢ Such significant rock mass mineralogical structure alterations certainly affect the aquifer’s flow characteristics, and aquifer permeability is enhanced by the long-term CO_2_ reaction in this study. For example, CO_2_ permeability at 4 MPa injection pressure and 20 MPa confining pressure increased by around 17% with CO_2_ saturation.
- ➢ The pore structure changes caused by the CO_2_ reaction also affect the effective stress response of the aquifer rock mass, and a significant rise in effective stress coefficient from around 0.95 to 3.5 was observed in this study.

## Additional Information

**How to cite this article**: Rathnaweera, T. D. *et al.* Experimental investigation of geochemical and mineralogical effects of CO_2_ sequestration on flow characteristics of reservoir rock in deep saline aquifers. *Sci. Rep.* **6**, 19362; doi: 10.1038/srep19362 (2016).

## Figures and Tables

**Table 1:** Mineralogical, chemical and geo-mechanical characteristics of the reservoir rock.

Lithology	Carbonate (CS) cementedsandstone
Formation	Early Triassic “Hawkesbury sandstone”
Macroscopic description	Colour: White-grey-brownish, medium to fine grained
Mineralogy (XRD)	60% quartz, 26% calcite, 6% kaolinite, 5% barite, 1% siderite, 1% muscovite, 1% other clay minerals
Structure	un-layered, inter-granular pore space
Cementation	Well cemented, sub-granular quartz rim with calcite fillings
Geochemistry (XRF, wt.%)	57.1 SiO_2_, 27.4 CaO, 4.3 Al_2_O_3_, 1.1 MgO, 5.4 Fe_2_O_3_, 3.3 K_2_O
Effective porosity (MIP)	29.4%
Permeability (mD)	94.32
Compressive strength (MPa)	48–52
Water absorption (%)	2.71

**Table 2:** Operating conditions for ICP-MS and ICP-AES tests.

ICP-MS	
Plasma conditions	
Rf frequency	28 MHz
Rf power	1.5 kW
Gas flow rate	
Carrier gas	Ar 1.01 min^−1^
Auxiliary gas	Ar 1.01 min^−1^
Coolant gas	Ar 16.0 min^−1^
Sampling conditions	
Sampling depth	10 mm from work coil
Sampling cone	Copper, 1.0 mm orifice diameter
Skimmer cone	Copper, 0.35 mm orifice diameter
Nebulizer	Glass concentric type
Sample uptake rate	0.7 ml/min
ICP-AES	
Plasma conditions	
Rf frequency	27 MHz
Rf power	1.0 kW
Gas flow rate	
Carrier gas	Ar 0.5 min^−1^
Auxiliary gas	Ar 1.01 min^−1^
Coolant gas	Ar 20.0 min^−1^
Sampling condition	
Nebulizer	Cross-flow type
Sample uptake rate	1.2 ml/min
Spectrometer conditions	
Polychromator	Paschen-runge mounting
Focal length	75 mm
Grating	2500 grooves mm^−1^
Entrance slit width	25 μm
Exit slit width	50 μm

**Table 3:** Chemical analysis of pure brine and brine taken from the reaction chambers and desiccator (concentration in mg/l).

Condition	pH	Na	K	Ca	Mg	Cl	Br	Si	Al	Fe	Mn	Ba
Initial brine	7.41	191433	2611	100.1	481	188311	1.43	0.11	—	—	—	1.11
Brine from desiccator after 1.5yrs	7.26	198304	3581	482.7	571	193110	1.50	874.11	—	—	—	13.26
Brine reacted with N_2_ after 1.5yrs	7.33	195917	3191	440.3	568	194215	1.42	815.31	—	—	—	12.21
Brine reacted with CO_2_ after 1.5yrs	4.81	277863	5868	3237	741	254782	1.63	4118	111	23.41	1.04	824.11

**Table 4:** Percentage of dissolved rock minerals in the three conditions with respect to natural mineral composition.

Rock mineral	Dissolved % with respect to initial composition		
	Pure brine(without gas)	Brine+N_2_	Brine+CO_2_
Quartz	0.95	0.88	4.49
Calcite	1.21	1.11	8.15
Barite	0.17	0.16	10.79
Siderite	—	—	1.53
